# Towards an environmentally sustainable and resilient healthcare system: a roadmap for Italy

**DOI:** 10.3389/fpubh.2026.1872869

**Published:** 2026-08-07

**Authors:** Ornella Punzo, Chiara Reno, Walter Cristiano, Rachel Juel, Laura Mancini, the SOSTERRETE collaborative group, Gabriella Abruzzo, Gloria Agazzi, Paola Angelini, Stefania Barcella, Francesco Barone Adesi, Katsiaryna Bashlakova, Sammy Bishop, Antonio Bonaldi, Giulia Bonanno, Sofia Borghi, Giuseppe Bortone, Roberta Bosco, Francesca Bravi, Serena Brunone, Eva Cappelli, Maria Assunta Cappelli, Mario Carere, Luisa Carracciuolo, Angelo Cieri, Walter Cristiano, Barbara Curcio Rubertini, Francesca De’ Donato, Manuela De Sario, Erica De Vita, Benedetta Dellisanti, Daniele D’Ettorre, Chiara Di Blasi, Domenico Di Fazio, Francesca Gorla, Vittorio Grieco, Laura Grisolia, Peppino Iannazzo, Rachel Juel, Ursula Kirchmayer, Paolo Lauriola, Jessica Lizzadro, Camilla Lugli, Aurora Mancini, Laura Mancini, Maricia Mancino, Marco Martuzzi, Roberta Monzani, Angela Nardin, Giovanni Nicolao, Lorenzo Nigi, Lia Olivo, Filippo Maria Panfili, Romina Pausilli, Alessia Pennimpede, Simone Priolo, Ornella Punzo, Francesca Racioppi, Laura Reali, Chiara Reno, Francesco Romizi, Roberto Romizi, Gianluca Santini, Behjat Shokati, Federica Tommasi, Chiara Tommasino, Francesco Traglia, Viola Turroni Casadei, Sandra Vernero, Giorgia Zanutto, Marianna Zarro, Salvatore Zimmitti

**Affiliations:** 1 Unit of Ecosystems and Health, Department of Environment and Health, Italian National Institute of Health, Rome, Italy; 2 Rete Nazionale per la Sostenibilità Ambientale del Sistema Sanitario (SOSTERRETE), Rome, Italy; 3 Evaluation Research and Health Services Policy, Romagna Local Health Authority, Ravena, Italy; 4 Scuola di Dottorato, Advances in Infectious Diseases, Microbiology, Legal Medicine, and Public Health Sciences, Sapienza University of Rome, Rome, Italy

**Keywords:** adaptation, climate change, health care, health systems, mitigation, policy, practice, roadmap

## Abstract

Healthcare systems play a dual role: they protect population health but also contribute to climate change through greenhouse gas emissions, resource use, and waste, while climate change is simultaneously increasing pressure on healthcare services through extreme weather events and rising disease burdens. In Italy, efforts to improve environmental sustainability and resilience in healthcare are growing but remain fragmented, with no comprehensive national framework. This paper presents a roadmap to support the commitment to a more sustainable and climate-resilient healthcare system, developed through an iterative participatory process with members of a national network of professionals (SOSTERRETE), including a national workshop hosted by the Italian National Institute of Health, followed by structured discussions and two rounds of asynchronous consultation. Contributions were synthesised to define shared principles, strategic objectives, priority actions, best practices, and policy recommendations. The findings identify key gaps in current policy and propose four strategic objectives: setting clear targets to reduce healthcare emissions, integrating sustainability into health system planning and governance, improving tools to measure environmental impacts and climate risks, and promoting actions that deliver both health and environmental benefits. Priority actions include improving energy use and infrastructure, reducing waste and unnecessary care, strengthening supply chain sustainability, enhancing preparedness for climate-related events, training healthcare staff, and establishing systems to track progress and ensure accountability. Italy has the chance to move from isolated initiatives to a coordinated national strategy, embedding sustainability into system governance, financing, and evaluation to reduce environmental impacts while improving resilience, health outcomes, and equity.

## 1 Rationale and context: why action is needed

This paper builds on a set of coordinated activities aimed at establishing an Italian nationwide network of experts and practitioners, focused on moving health systems towards environmental sustainability and at strengthening technical capacity in this sector. These activities started in 2025 with a national workshop hosted by the Istituto Superiore di Sanità (the Italian National Public Health Institute, ISS) whose main result was establishing a network of professionals working in this field. The roadmap presented in this paper is a direct output of structured inputs and contributions collected during the workshop and subsequent activities. This work includes developing a distance-learning module on environmental sustainability in health systems, organising an interactive session on health system decarbonisation for COP30 in Belém, Brazil, and finally a hybrid event on pathways towards decarbonised and resilient health systems globally in March 2026, co-hosted by the ISS and the Romagna Local Health Authority, with the support of SOSTERRETE (the newly built national environmental sustainability network) and under the auspices of the World Health Organization (WHO).

## 2 Healthcare and climate change: evidence, impacts, and policy gaps

While protecting population health, the healthcare sector is itself a significant contributor to greenhouse gas (GHG) emissions, which are the primary driver of climate change (1). Globally, the sector accounts for approximately 4.4–6% of net GHG emissions (2, 3), equivalent to an estimated 523 kg CO₂e per capita, based on 2018 data (4). If it were a country, the global healthcare sector would rank as the fifth-largest GHG emitter worldwide (5). In health systems, emissions are distributed across 3 types of sources: direct emissions (scope 1), indirect energy emissions (scope 2), and other indirect emissions (scope 3) as defined below (4):

- Scope 1: direct emissions from sources owned or controlled by healthcare entities, including anesthetic and medical gases (approximately 12% of sectoral emissions (4);
- Scope 2: indirect emissions from purchased electricity, heat, or steam (approximately 9%) (4);
- Scope 3: all other indirect emissions across the healthcare value chain, including resource intensive-procurement and supply chains, transport, outsourced services, and waste management (approximately 79%) (4).

At the same time, the effects of climate change are increasing demand for healthcare services. Evidence indicates that a 1 °C rise in average temperature would be associated with an approximate 4.5% increase in hospital admissions (6). Climate-related extreme events, including floods, heatwaves, and wildfires, further disrupt healthcare delivery by damaging infrastructure, disrupting supply chains, and straining workforce capacity (7, 8). In Italy, the healthcare sector is estimated to contribute about 4% of national GHG emissions, underscoring its relevance to national climate mitigation efforts (9, 10); this figure is consistent with more recent OECD estimates showing that health systems account, on average, for 4.4% of demand-based greenhouse-gas emissions (GHGEs) across OECD countries (11). The main sources of emissions include hospital care, energy use in healthcare facilities, pharmaceuticals, medical devices. and other supply-chain-related emissions (11). OECD analyses indicate that hospitals account for around 30% of health-sector emissions across OECD countries, while supply chains are responsible for nearly four-fifths of the sector’s total emissions, underscoring the importance of addressing both direct operational emissions and indirect emissions embedded in procurement and care pathways (11).

The Italian National Recovery and Resilience Plan (PNRR), through Missione 6 Salute, allocated €15.63 billion to health, with additional complementary national resources. These investments have primarily targeted the reorganisation of territorial care, including Case della Comunità (Community Houses), home care and telemedicine, and Ospedali di Comunità (Community Hospitals), together with the digital and technological modernisation of hospitals, including large diagnostic equipment, digitalisation of emergency departments, and additional intensive and semi-intensive care capacity (12). Other relevant measures include investments in safer and more sustainable hospitals, research, training, and programmes linking health, environment, biodiversity and climate. However, these investments were not designed as part of a dedicated healthcare decarbonisation strategy and do not yet provide a systematic framework for measuring, reducing and monitoring the carbon footprint of healthcare services. What remains missing is therefore additional funding, and a coherent governance framework. Italy still lacks a dedicated national roadmap for low-carbon and climate-resilient healthcare, routine carbon accounting at national, regional and organisational levels, mandatory or incentivised green procurement criteria for pharmaceuticals, medical devices and services, integration of carbon indicators into quality and performance monitoring, and explicit mechanisms to align PNRR-funded reforms with healthcare-sector mitigation objectives. Addressing these gaps would allow existing investments in territorial care, digitalisation, infrastructure and resilience to become part of a measurable transition towards a sustainable healthcare system.

In response to these challenges, environmental sustainability and resilience of healthcare systems have become central topics in international policy and scientific discourse. The WHO defines climate-resilient health systems as those capable of anticipating, responding to, coping with, recovering from, and adapting to climate-related shocks and stresses, thereby achieving sustained improvements in population health, despite an unstable climate (13). Global initiatives, including the COP26 Health Programme and the WHO Alliance for Transformative Action on Climate and Health (ATACH) (14), have emphasised the need to simultaneously reduce healthcare-related emissions and integrate climate considerations into health system planning and governance.

To date, more than 100 countries have committed to developing climate-resilient, low-carbon health systems (15, 16). However, within the WHO European Region, only 10 of the 53 countries have formally committed to strengthening health system climate resilience and/or reducing healthcare-related GHG emissions, indicating a substantial implementation gap (17).

The reduction of healthcare’s environmental footprint has also been advanced by non-governmental organisations (NGOs), notably Healthcare Without Harm (HCWH), an international civil-society network that supports healthcare decarbonisation and environmentally sustainable healthcare through advocacy, technical guidance, and institutional networks. In Europe, HCWH promotes environmentally sustainable healthcare practices through policy engagement, technical guidance, and capacity building (18).

Taken together, these initiatives underscore the urgent need to accelerate the transformation of healthcare systems towards climate resilience and environmental sustainability. In this context, environmental sustainability should become a guiding principle for health policy, shaping planning, investment, and service delivery across the health system. This requires integrated approaches that combine emission mitigation, climate adaptation, and improvements in system-wide environmental performance, supported by cross-sectoral collaboration and interdisciplinary approaches.

### 2.1 The Italian healthcare service: governance and financing

The Italian National Health Service (Servizio Sanitario Nazionale, SSN) was established in 1978 (Legge 23 dicembre 1978, n. 833) (19) and, similar to the UK NHS, it is publicly funded through the national and regional tax system. The SSN is universal, i.e., it provides comprehensive healthcare access to all residents regardless of their income. Although the central government defines the Essential Levels of Care (LEA, Livelli Essenziali di Assistenza) to ensure consistency across the whole country, allocates national funding, and sets the overall regulatory framework, responsibility for healthcare planning, organisation, and service delivery is largely devolved to the regional health systems through local health authorities and public and accredited private providers (20). This level of regional autonomy was enshrined in 2001 by a constitutional amendment (Constitutional Law N. 3 of 18 October 2001: Amendments to Title V of Part II of the Constitution) (30). This constitutional reform, which governs the territorial organisation of the Republic, the regions, the provinces, and the municipalities by delegating the organisation and management of health services to the regions and autonomous provinces, aimed to foster a form of federalism based on solidarity. However, it has ultimately led to a drift towards regionalism, resulting in 21 different health systems where access to health services and care varies greatly and is inequitable (21). This governance model has important implications for environmental sustainability. The high degree of regional autonomy has resulted in substantial variability in infrastructure, investment capacity, environmental priorities, and data collection systems across the country (22). The Italian healthcare system is also highly resource- and energy-intensive. This is mainly driven by regional fragmentation, decentralized purchasing, overused medical tests, and poor local care (12). Moreover, a large share of hospital infrastructure is ageing and was not designed according to modern energy-efficiency standards. Consequently, sustainability initiatives have often developed locally rather than within a coordinated national framework, limiting comparability, the adoption of common indicators, and the implementation of uniform decarbonisation strategies. Addressing these challenges requires coordinated governance, harmonised monitoring systems, and long-term investment strategies capable of supporting both mitigation and adaptation across all regional health systems (22).

### 2.2 International implementation frameworks

Several countries have already translated international commitments into national implementation frameworks for sustainable healthcare. In England, the Greener NHS programme combines legally supported net-zero targets, mandatory Green Plans, and a national monitoring framework (23). In the Netherlands, the Green Deal on Sustainable Healthcare 3.0 provides a collaborative national framework involving the central government, healthcare providers, insurers, professional associations, research institutions, and industry (Green Deal Sustainable Healthcare 3.0) (24). The initiative establishes shared objectives for carbon reduction, circular resource use, sustainable procurement, prevention, and pharmaceutical pollution, while supporting implementation through coordinated action across the health sector. Similar national strategies have also been developed in countries such as Canada and Australia, although they differ in governance models and implementation mechanisms (25, 26). By contrast, Italy has not yet adopted a comparable nationwide implementation framework, and most sustainability initiatives remain regional or locally driven.

## 3 Approach and guiding principles

This paper adopts an integrated approach to environmental sustainability and climate resilience in health systems, recognising that health outcomes, climate risks, and environmental impacts are shaped by decisions across multiple policy domains and governance levels. Climate change affects health systems by increasing demand for healthcare services and by disrupting infrastructure, supply chains, and workforce capacity. Responding effectively to these interconnected challenges requires a coherent policy framework that integrates climate mitigation, climate adaptation, and the generation of health co-benefits, and that is operationalised through clearly defined guiding principles.

### 3.1 Health in all policies

Health considerations should be systematically integrated into public policies at national, regional, and local levels. This principle is firmly grounded in European Union law: Article 168 of the Treaty on the Functioning of the European Union (TFEU) requires that a high level of human health protection be ensured in the definition and implementation of all Union policies and activities (27).

### 3.2 Climate resilience through integrated mitigation and adaptation

Climate resilience requires joint consideration of climate mitigation and adaptation. Measures that reduce GHG emissions from healthcare delivery, such as energy efficiency, sustainable procurement, and low-carbon service models, help reduce long-term climate risks that would otherwise increase pressure on health systems. At the same time, adaptation measures to strengthen infrastructure, service continuity, and emergency preparedness must be designed to avoid increasing emissions or environmental burdens. Mitigation and adaptation should therefore be understood as complementary and mutually reinforcing components of climate resilience. Treating them in isolation risks undermining long-term system sustainability and missing opportunities to generate co-benefits for health, the environment, and the economy.

### 3.3 Cross-sectoral, interdisciplinary, and multi-stakeholder action

The climate crisis demands systemic responses that extend beyond the health sector. Effective climate mitigation and adaptation depend on coordination across health, environment, energy, transport, water, waste, urban planning, and civil protection systems, as well as engagement with academic institutions, civil society, private-sector actors, and communities. Interdisciplinary and multi-stakeholder approaches mobilise diverse expertise, resources, and perspectives needed for complex system transformation.

Recent climate-related emergencies have underscored the importance of such coordination. The 2023 floods in the Emilia-Romagna Region (Italy) showed that effective preparedness, response, and recovery depend on close collaboration among healthcare services, emergency responders, local and regional authorities, infrastructure operators, and community organisations (79).

Strengthening these collaborative mechanisms is not only essential for emergency response but also for long-term planning to reduce vulnerability and enhance the environmental sustainability and resilience of health systems.

## 4 Methodology

This manuscript was conceived as an exploratory, evidence-informed, and participatory roadmap developed through a structured expert consultation, not as a primary empirical study. Its content was developed through a hybrid national workshop, structured participatory discussions, and two rounds of asynchronous consultations aimed at consolidating shared principles, strategic objectives, priority actions, and policy recommendations. As this paper is the result of the work of the first formal national network dedicated to promoting and sharing best practices in the environmental sustainability of healthcare, and because this paper represents its first output, the initiative was necessarily conceived as a baseline-building exercise.

The workshop aimed to initiate the development of a roadmap and baseline paper to promote sustainable practices in healthcare systems, with a specific focus on reducing the sector’s carbon emissions and identifying actionable strategies for mitigation and climate resilience. The one-day workshop entitled “Towards a network for the sustainability of the healthcare system” (“Verso la rete per la sostenibilità del sistema sanitario”) was organised by the Unit of Ecosystems and Health of the Department of Environment and Health of the Italian National Institute of Health (Istituto Superiore di Sanità, ISS) on 18 June 2025. The event was held in a hybrid format at ISS in Rome and online via Microsoft Teams. For this first event, we invited stakeholders from several networks, involving professionals from health and research institutions with documented experience, project involvement, or scientific production in the field of healthcare sustainability. We invited experts who joined the first national conference of Sistema Nazionale Prevenzione Salute dai rischi ambientali e climatici (SNPS—National Health Protection System against Environmental and Climate Risks), that took place 27–28 March 2025 (28), lecturers of the PNC (Piano nazionale complementare—complementary national plan) SABiC (National training programme in Salute-Ambiente-Biodiversità-Clima (SABiC)—Health, Environment, Biodiversity and Climate of Istituto Superiore di Sanità) distance learning module “La sostenibilità ambientale dei sistemi sanitari” (The environmental sustainability of health systems) which was launched in November 2025 on the Eduiss website, colleagues working on contiguous projects at the Department of Epidemiology of the Lazio Regional Health Service—ASL Roma 1 (DEP Lazio), ISDE (International Society of Doctors for the Environment) network, colleagues and public health medical residents from University Sapienza, besides all colleagues at ISS. Documentation procedures included participants’ registration, distribution of informational materials, input collection during plenary and workshop discussions, and post-event gathering of written feedback. Attendance was monitored through in-person registration records and online connection logs. Forty-four people registered in person and 47 remotely.

The programme combined expert presentations, plenary discussion, and group work. Invited speakers represented national and international public health, academia, healthcare, and civil society perspectives. Contributions included representatives from the WHO Regional Office for Europe, Greener NHS/NHS England, Healthcare Without Harm, the Global Green and Healthy Hospitals network experience in Italy with Azienda USL Romagna, CRIMEDIM and the University of Piemonte Orientale, and ISS. The scientific framing addressed the health impacts of climate change, the role of healthcare systems in the climate crisis, decarbonisation strategies, international experiences in transitioning to net-zero healthcare, and the integration of mitigation and adaptation for climate-resilient health services. The morning sessions were structured around expert presentations followed by question-and-answer sessions and interactive plenary discussions. The afternoon workshop focused on collaboratively identifying priority actions to reduce healthcare-sector emissions in the Italian context. The workshop setting was designed to privilege direct interaction among in-person participants; additional written contributions from online participants were welcomed by email after the event.

Participant characteristics were assessed by integrating the event registration database with the in-person attendance records and the Microsoft Teams attendance report. Forty-five unique participants were identified, of whom 23 attended in person and 22 online. Registration information was available for 44 participants and was used to assess geographical distribution, institutional affiliation, seniority, and previous experience in healthcare environmental sustainability or related fields. Geographical representation was assigned based on the location of each participant’s declared institution, rather than their place of birth or residence, as this was considered a more appropriate indicator of the event’s territorial and institutional reach. Institutions that were national or international in scope, not geographically identifiable or not reported, were classified separately. Overall, 22 of the 45 participants were affiliated with institutions in Lazio, accounting for approximately 49% of the total. The in-person group was particularly concentrated in the host region, whereas online participation broadened the geographical reach to Emilia-Romagna, Lombardy, Veneto, Tuscany, Piedmont, Campania, and other areas. Nevertheless, the overall distribution remained weighted towards Lazio and central and northern Italy, while southern regions and the islands were under-represented. Institutional affiliations were grouped into broad categories, including academic institutions, public health bodies, healthcare providers, regional and local health authorities, non-governmental organisations and technical or professional organisations. Academic institutions represented the largest category, particularly among in-person attendees. The online component increased institutional diversity by involving participants from local health authorities, hospitals, Scientific Institutes for Research, Hospitalisation and Healthcare (IRCCS), regional public health services, non-governmental organisations and technical or engineering backgrounds. However, representation from procurement services, facility management, hospital executive leadership, regional health planning, private or accredited healthcare providers, and patient or citizen organisations remained limited. Seniority was assessed based on professional roles and career stages. The in-person group included a substantial proportion of early-career professionals and postgraduate public health participants, whereas the online group included a higher proportion of senior and operational professionals. The resulting participant group, therefore combined an important capacity-building and network-development component with input from professionals holding greater decision-making, technical or implementation responsibilities. Previous experience was classified based on the professional role and information provided during registration. Approximately half of the integrated participant group had direct, closely related or broader experience in healthcare sustainability, environmental health or sustainability-related activities. Specific areas of expertise included hospital sustainability, green anaesthesia, energy and mobility management, environmental pharmacoepidemiology, regional environment-and-health activities, environmental health networks and healthcare sustainability committees. The remaining participants were mainly early-career public health professionals, clinicians or other professionals with a general interest in the topic but limited topic-specific experience. Overall, the composition of the group supports the interpretation of the event as a hybrid network-building and capacity-building exercise, enriched by selected senior and operational expertise, rather than as a nationally representative consultation or formal expert consensus process.

Following this event, SOSTERRETE was launched, a network of experts, public health officials, physicians, academics, and civil society representatives working on the environmental sustainability of health systems.

The process was conceived as iterative. Following the workshop, experts involved in the emerging network were invited to provide further comments, recommendations, and revisions to the draft document, to develop a final paper as part of a broader roadmap to guide healthcare professionals, patients, and policy-makers in the implementation of sustainable and low-carbon healthcare strategies.

The content generated during the workshop discussions was synthesised by the two researchers who facilitated the sessions. Their notes were collated and jointly reviewed, and the main contributions were organised into bullet points grouped under thematic areas. On this basis, a first draft of the paper was prepared and circulated to all contributors for asynchronous consultation.

The consultation was conducted through two successive rounds of written review. During each round, contributors were invited to comment on the accuracy, relevance and completeness of the text, propose revisions and additions, and identify missing perspectives or examples. All comments were collated by the coordinating authors in a consolidated working document and reviewed individually. Contributions were incorporated either directly or through synthesis when consistent with the scope, evidence base and overall coherence of the paper. Overlapping comments were merged, while there were no competing or conflicting suggestions. Where proposals could not be incorporated verbatim, their substantive content was retained through reformulation or integration into broader statements.

The process was intended to capture the breadth of potential intervention targets, strategic areas and recommended actions relevant to the environmental sustainability and climate resilience of the Italian healthcare system. It was not designed as a formal prioritisation exercise, and the proposed actions were not ranked according to importance, feasibility, cost-effectiveness, or expected impact.

Following the first consultation round, the revised draft was recirculated so that contributors could review the amendments and the integration of earlier comments. The second round focused on resolving remaining inconsistencies, refining the wording, strengthening under-represented areas and agreeing on the final framing and recommendations. Final editorial decisions were made by the main authors, with disagreements addressed through discussion and iterative revision rather than through formal voting or predefined consensus thresholds. All contributions were reviewed and incorporated, either directly or through synthesis, where compatible with the paper’s scope and internal coherence.

### 4.1 Limitations

A key methodological limitation stems from the absence of any pre-existing national platform, public body, or established reference framework through which relevant stakeholders could be systematically identified and engaged. The contributors, therefore, constitute a purposively engaged, self-selected group of relevant stakeholders rather than a nationally representative group. Achieving national representativeness was not feasible, particularly given the highly decentralised organisation of the Italian healthcare system since the 2001 constitutional reform (30), with responsibilities, expertise, and sustainability initiatives distributed across regional and local levels.

The process was conceived as a participatory, expert-informed drafting and network consultation exercise rather than formal research or consensus-building methodology. No mixed-methods study, interviews, focus groups, qualitative coding, thematic analysis, Delphi process or other structured consensus procedure was undertaken. Contributions generated during the workshop were not assigned differential weights according to participants’ seniority, professional role or degree of topic-specific expertise. It was therefore not possible to distinguish systematically between the influence of highly specialised experts and that of participants with broader, related or emerging expertise. Consequently, the collection and synthesis of contributions may have been affected by subjectivity, unequal participation, and the judgement of the coordinating authors. The roadmap should not be interpreted as a ranked set of priorities. The participatory process did not include predefined prioritisation criteria, comparative scoring, formal stakeholder weighting, economic appraisal or a structured consensus method. Consequently, the recommendations describe a broad range of potentially relevant actions rather than establishing their relative importance, sequencing or expected magnitude of impact. Further empirical and decision-analytic work will be required to prioritise interventions across national, regional and organisational contexts.

The resulting paper should therefore be interpreted as a collaboratively developed position statement reflecting the perspectives of a self-selected and heterogeneous group of stakeholders, rather than as the outcome of a formal empirical study, a nationally representative consultation or a weighted expert consensus process and as an initial reference framework to support future, scientifically rigorous, and more extensive evaluation.

## 5 Strategic objectives for the Italian NHS

Italy has recently strengthened the integration of health, environment, biodiversity, and climate policies through the adoption of the 3 years-long Programma Salute, ambiente, biodiversità e clima (National Health, Environment, Biodiversity and Climate Programme) (29) through the establishment of the Sistema Nazionale di Prevenzione Salute (SNPS—National Health Prevention System) to protect human health from environmental and climate-related risks (30) and through the 2026–2031 Piano Nazionale della Prevenzione (PNP—National Prevention Plan) including a focus on environment, climate and health, among a total of 7 priority areas (31).

Nevertheless, Italy has not yet joined the WHO ATACH, and therefore, has not formally committed to the goal of developing low-carbon health systems or to achieving climate neutrality in healthcare by 2050. At present, no nationally defined targets or timelines exist for the decarbonisation of the Italian healthcare sector, nor is there a comprehensive framework to guide emission reductions across healthcare delivery and supply chains (9, 32).

The Italian National Institute of Health has identified participation in this international network as a strategic opportunity to further integrate climate and health within the broader national mitigation and adaptation agenda. Joining ATACH would provide Italy with access to internationally recognised methodologies, technical guidance, peer learning, and collaborative networks while strengthening governance through shared monitoring approaches and accountability. As demonstrated by countries that have already adopted national implementation frameworks, such as England through the Greener NHS programme and the Netherlands through the Green Deal Sustainable Healthcare, a formal national commitment can provide the governance structure needed to translate strategic ambitions into measurable actions. In this regard, the roadmap proposed in this paper is intended as a first step towards aligning the Italian healthcare system with these emerging international best practices while accounting for the specific governance characteristics of the Italian NHS.

**Figure 1:**
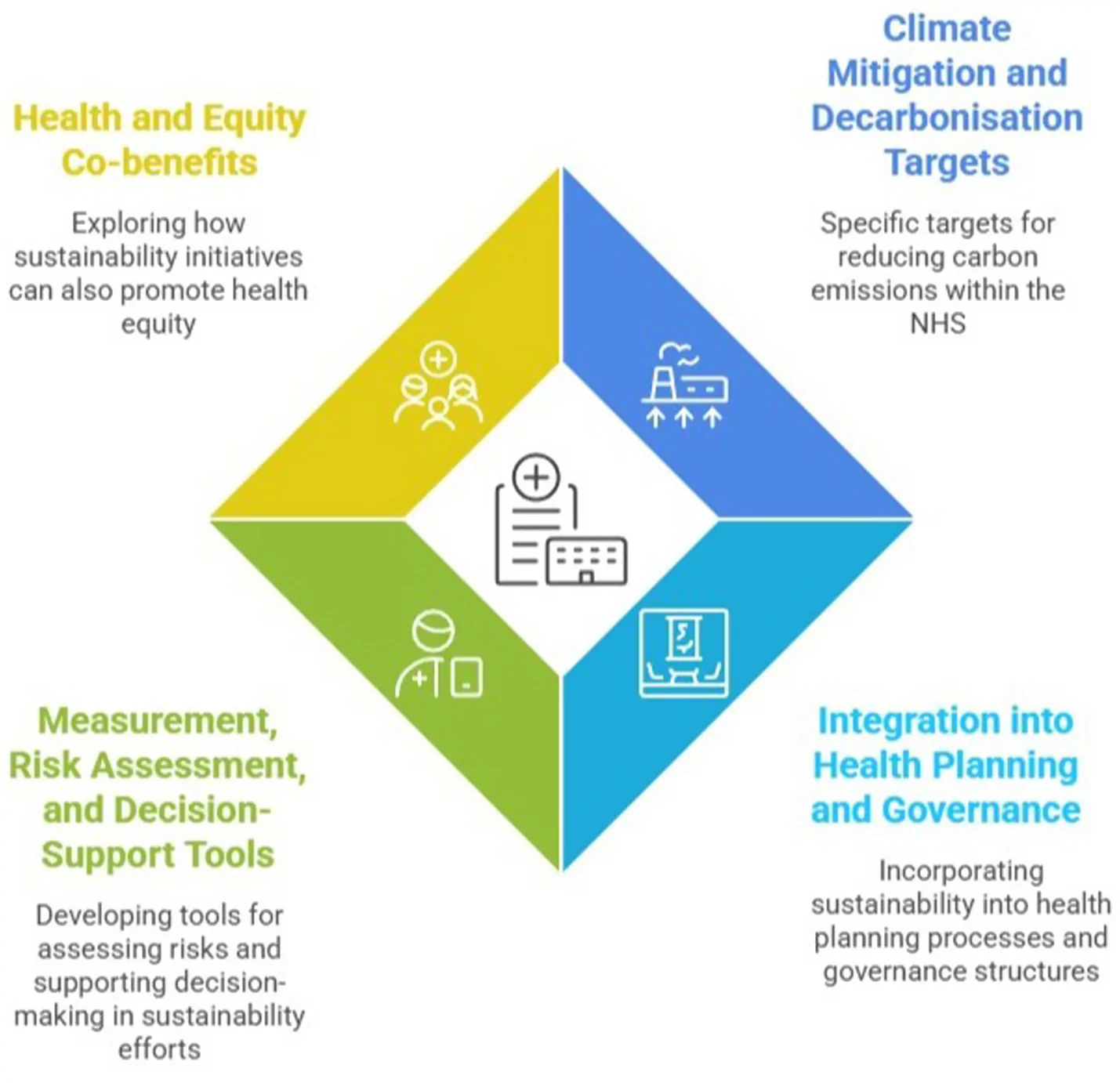
Strategic objectives for an environmentally sustainable Italian National Health Service (80).

In this context, the primary objective of this strategic roadmap is to support Italy’s commitment to achieve climate neutrality. in the short term, building the political and institutional conditions required to pursue it. The following strategic objectives (Figure 1) are proposed to support this commitment. These objectives are intended to align mitigation and adaptation efforts, strengthen accountability, and enable the generation of health and equity co-benefits.

### 5.1 Climate mitigation and decarbonisation targets

Targets for climate mitigation decarbonisation have been adopted by the English National Health Service (NHS), which committed to decarbonisation in 2020 with the ambition to reach an 80% reduction in emissions between 2028 and 2032, relative to 1990 baseline. The NHS aims to reach net zero for Scope 1 and 2 emissions, which they can control, by 2040 and net zero for the wider health system footprint, encompassing Scope 1, 2, and 3, by 2045 (23). The NHS’s commitment was enabled by the presence of national and sub-national leadership, resources, and capacity, with dedicated sustainability leads and teams increasingly established across NHS trusts (33). A more realistic comparator for Italy is Belgium, which developed a national healthcare decarbonisation roadmap and established a 2022 emissions baseline, but has not yet adopted a definitive emissions-reduction target for the health sector (34).

Building upon these existing commitments, the primary objective of this roadmap should be for public hospitals to commit to the achievement of 80% reduction of Scope 1 and Scope 2 emissions by 2040, at the latest. The SSN, as a whole, should work towards net zero across the healthcare value chain, including Scope 3 emissions, by 2046.

This distinction between Scope 1, 2, and 3 is important because healthcare organisations directly control most Scope 1 emissions and can substantially influence Scope 2 emissions, whereas Scope 3 emissions depend largely on suppliers, manufacturers, procurement systems, and wider markets. Individual organisations can influence Scope 3 through demand reduction and purchasing decisions, but achieving substantial reductions requires coordinated national and regional procurement policies, supplier requirements, and industry action.

These dates recognize the delay of six or more years from the commitment made by the UK in 2020 and 4 years delay from the commitment made by Belgium in 2022. As such, the proposed timeline of commitment resulting from this roadmap by Italy, is proportionate to the inherent delay, and may need to be adjusted to the country’s existing capacity and commitment date. An interim objective could additionally be to reduce healthcare-related emissions as rapidly and substantially as possible, consistent with pathways that are compatible with limiting global warming to 1.5 °C.

### 5.2 Integration into health planning and governance

Environmental sustainability should be systematically integrated into national, regional, and local health planning processes. This includes embedding climate mitigation objectives within health system governance, investment decisions, and service organisation, and ensuring coherence with broader environmental and climate policies.

In parallel, climate adaptation and health system resilience should be explicitly incorporated into national and regional prevention programmes (35). Strengthening preparedness for climate-related shocks—such as heatwaves, floods, and supply chain disruptions—supports continuity of care and reduces long-term system vulnerability.

### 5.3 Measurement, risk assessment, and decision-support tools

Achieving these objectives requires widespread adoption of tools to measure healthcare-related emissions and assess environmental impacts. These include carbon accounting instruments, e.g., Climate Impact Check-up tools, and the integration of life-cycle assessment approaches within Health Technology Assessment (HTA) processes (36).

In addition, health systems should adopt tools to assess climate-related vulnerability, risk, and preparedness at the facility and system levels. Instruments such as vulnerability and preparedness checklists for healthcare facilities can support risk identification, prioritisation of adaptation actions, and targeted investment (37).

### 5.4 Health and equity co-benefits

Finally, strategic actions should prioritise interventions that generate co-benefits for population health, health equity, and environmental sustainability. Integrating mitigation and adaptation measures can reduce climate-related health risks, improve air quality and working conditions, enhance system efficiency, and support more equitable access to care. Explicitly recognising and monitoring these co-benefits can strengthen the case for sustained investment in climate action within the health sector.

## 6 Priority actions for implementation

**Figure 2:**
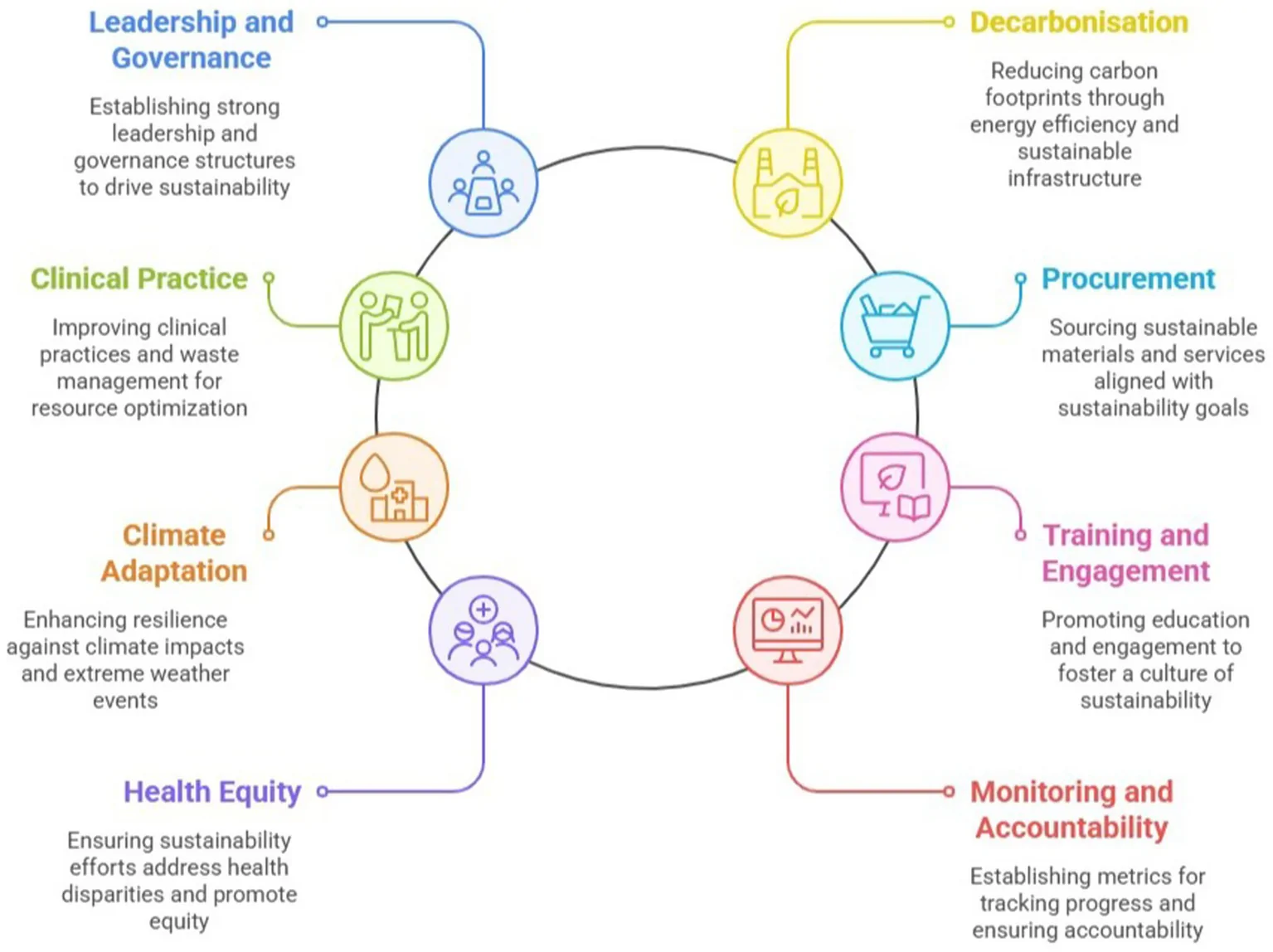
Priorities for environmentally sustainable healthcare.

To build a resilient and environmentally sustainable healthcare system, priority actions should span multiple domains and address both climate change mitigation and adaptation. The following sections outline a comprehensive set of actions across the following concepts: leadership, decarbonisation, procurement, clinical practice, adaptation, training, and co-benefits (Figure 2).

### 6.1 Leadership and governance

Strong leadership is critical to the success of climate action in the healthcare sector. This includes both the leadership role of health systems in advancing climate action across other sectors and clear internal leadership within healthcare organisations. The Global Roadmap for the Decarbonization of Healthcare emphasises that the health sector, by drawing on its ethical, political, and economic authority, can exercise meaningful leadership in the societal response to climate change (38). WHO’s operational framework for building climate-resilient and low-carbon health systems aims to support the design of transformative health systems that can provide safe, high-quality care in a changing climate (8). Owing to its mandate to safeguard population health, the sector can play a pivotal role in promoting climate resilience and advancing health equity. Through a range of strategic levers, healthcare systems can contribute to progress that extends beyond the commitments made by individual countries under the Paris Agreement, thereby supporting a more rapid transition towards clean energy and a low-emission, climate-responsive model of care. These levers could be pulled through strategic actions. National and regional health authorities should establish clear governance structures to coordinate climate action across healthcare institutions, ensuring accountability and alignment with broader national and regional climate goals. Leadership training should be promoted among senior healthcare administrators to integrate sustainability into health policy and decision-making. Mandatory Green Plans for all public healthcare entities should be developed and implemented to ensure systematic climate action. Sustainability criteria should be incorporated into healthcare accreditation and performance evaluation, rewarding healthcare institutions that lead in climate action and environmental sustainability. Access to adequate funds and the development of effective financing mechanisms to support the transition towards sustainability across the healthcare sector should be ensured.

### 6.2 Decarbonisation of healthcare operations and infrastructure

To address the healthcare sector’s emissions, significant decarbonisation across operations and services is essential. Key actions should focus on reducing direct emissions from healthcare institutions and integrating sustainability into service delivery.

- Calculate and manage GHG emissions: Healthcare organisations should calculate their Scope 1, 2, and 3 emissions and develop comprehensive carbon management plans. These plans should be guided by clear, measurable, and time-bound intermediate objectives, as suggested by ATACH’s guidance for measuring greenhouse gas emissions in health systems (39).
- Energy and Infrastructure Management: Health systems should reduce energy consumption, implement energy efficiency measures, and transition to renewable energy sources. Local experiences, such as those of the Romagna Local Health Authority, can provide valuable insights into achieving these goals (40). By reducing energy demand and dependence on imported fossil fuels, healthcare decarbonisation and renewable-energy deployment can limit exposure to geopolitically driven supply disruptions and energy-price volatility, thereby strengthening the financial and operational resilience of health services (41).
- Design and implement resilient infrastructure that meets modern environmental and climate criteria, ensuring it can withstand climate shocks and support service continuity during extreme events (8).
- Improve building energy management by integrating smart systems that optimise heating, cooling, and lighting, reducing energy consumption and operational costs (42).
- Effective water saving and good water management in hospitals are crucial for both environmental sustainability and operational efficiency. This directly reduces GHGEs by lowering the energy required to pump, heat, treat, and distribute water and to treat wastewater (8).
- Reduce high-GWP drug and device use: replace high-global warming potential drugs and devices, such as desflurane and other anaesthetic gases, with more sustainable alternatives. This can significantly reduce emissions from anaesthesiology, which has one of the highest carbon footprints in healthcare (43). In the operating room, it is possible to promote the limitation or abolition of desflurane, as has already been done in other European countries (44, 45), and to promote the reduction of halogenated gas consumption by using low-flow administration or alternative techniques of equivalent efficacy. Particular attention should also be given to the surgical pathway, which is responsible for emissions three to six times higher than in other hospital sectors (46). This principle also applies to some respiratory inhalers, particularly pressurised metered-dose inhalers (pMDIs), that use high-GWP propellants. Optimising inhaler choice and use (including correct technique and adherence) can improve asthma control and reduce exacerbations, while also reducing the environmental footprint of care. The Global Initiative for Asthma provides guidance on selecting the “optimal inhaler” for both patient outcomes and sustainability (47), and the NICE asthma pathway similarly highlights the environmental impact of different inhaler devices (48). A key European reference for anaesthesiology is the 2023 Glasgow Declaration, promoted by the European Society of Anaesthesiology and signed by nearly all national scientific societies, including the Italian Society of Anaesthesia, Analgesia, Resuscitation, and Intensive Care (SIAARTI). Additionally, in the EU, the revised fluorinated greenhouse gas (F-gas) Regulation prohibits the use of desflurane as an inhalation anaesthetic from 1 January 2026, except where it is strictly required on medical grounds (44). Despite this, Italy is one of the few European countries where desflurane use did not reduce in the last few years (49), and there remains more than 20-fold difference in per capita desflurane use across Italian regions suggesting that there is room for a substantial reduction in the use of this gas, while maintaining high-quality care (50). The marked regional variation in per capita desflurane use, illustrates how clinical practice, procurement choices and organisational cultures can translate into very different environmental impacts within the same national health system. Such variation suggests that decarbonisation cannot rely only on infrastructure investments, but requires comparable indicators, national guidance, professional engagement, and mechanisms to identify and scale low-carbon practices across regions.
- Limit nitrous oxide use in hospitals: nitrous oxide should be restricted to selected clinical cases, with centralised distribution systems phased out. Previous studies have reported that 75–95% of nitrous oxide supplied through hospital pipeline systems may be lost before clinical use, with some audits recording losses above 90% (51, 52). Administering the gas via cylinders only in the few clinical settings where necessary significantly reduces the carbon footprint of this gas, which has an atmospheric half-life of over a century. As proof, new hospitals under construction often no longer include nitrous oxide distribution lines. However, this approach should explicitly account for nitrous oxide–oxygen mixtures commonly used for analgesia (e.g., procedural pain relief, labour), where any restriction must be paired with clear clinical alternatives and protocols to maintain pain control and patient safety (for example, other pharmacological and non-pharmacological analgesic options appropriate to the setting). As proof, new hospitals under construction often no longer include nitrous oxide distribution lines, instead relying on targeted point-of-care provisions where clinically justified and supported by agreed alternative analgesia pathways.

### 6.3 Clinical practice, service organization, and waste management

Healthcare settings, particularly hospitals and operating rooms, contribute substantially to healthcare emissions. Clinical practices and waste management systems must be redesigned to mitigate environmental impact.

- Reduce special waste in operating rooms: Waste audits show that substantial quantities of operating-room waste (up to 64%) can be managed through ordinary or recyclable waste streams rather than as biohazardous waste (53, 54). Train staff in waste segregation and introduce systems to identify which waste can be recycled or diverted from incineration, leading to both environmental and cost savings.
- Adopt circular economy models for waste management: implement AI-driven tools for managing hospital waste, helping institutions optimise recycling and reduce environmental impact while supporting Scope 3 emissions targets (55). Where available, this should also include reuse and remanufacturing practices (e.g., reprocessing eligible devices and adopting reusable alternatives) to reduce waste generation upstream and further lower environmental impacts.
- The digitalisation of healthcare and the wider use of telemedicine are increasingly recognised as practical ways to reduce emissions, largely by reducing the need for travel and some on-site activities. Several studies reviewed by Braithwaite et al. (56) show that virtual consultations can meaningfully lower transport-related emissions while preserving care quality, and that reorganising follow-up pathways or relocating certain services closer to communities can further limit unnecessary mobility. When combined with improved scheduling, better logistics, and attention to equity in access to digital services, these measures offer a concrete and feasible contribution to the broader decarbonisation of health systems.
- Clinical appropriateness—The monitoring of appropriateness, particularly with regard to overuse, is seen as one of the most important measures for mitigating the carbon footprint of healthcare services. A frequently cited synthesis estimates that only 60% of treatment is based on guidelines of recognised effectiveness, while 30% are of little to no clinical value and 10% are actually harmful (57).
- Reducing low-value care, waste, and unnecessary hospital time could cut hospital-related emissions by up to 25% on average across OECD countries, according to modelled estimates (4). Promoting appropriate care not only improves health and lowers costs but also benefits the environment, reinforcing the case for reducing low-value interventions. Also, avoiding inappropriate medication use through targeted interventions on appropriate drug prescribing such as medication review/ deprescribing can reduce waste from drugs and indirectly reduce access to health care as a consequence of drug related adverse events (e.g., drug–drug interactions, falls).
- Several international initiatives have emerged to address this issue, including Choosing Wisely, launched in the United States in 2012 and now active in 35 countries. In Italy, it is led by Slow Medicine through the campaign “Doing more does not mean doing better” (58), which includes numerous green recommendations and is supported by 50 + national professional societies that have issued 300 + recommendations on unnecessary tests, treatments, and procedures

### 6.4 Procurement and healthcare supply chain

Given the dominance of Scope 3 emissions, national and regional procurement authorities should establish common environmental requirements for suppliers, while individual healthcare organisations should implement these requirements through purchasing decisions and demand reduction. Therefore, healthcare systems must leverage their purchasing power to drive sustainability in their supply chains.

- Extend Minimum Environmental Criteria (CAM in Italian) to include green procurement strategies in all health procurement decisions, prioritising environmental sustainability in sourcing of products and services and requiring suppliers to have corporate targets and plans to decarbonise their operations, in line with the recommendations of the WHO guidance Decarbonizing the healthcare supply chain: strategic actions for health systems (15).
- Incorporate climate resilience criteria in procurement contracts, ensuring that suppliers meet specific standards for both mitigation and adaptation.
- Reduce unnecessary mobility and transportation by promoting local and diverse supply chains to strengthen resilience and reduce emissions associated with transportation and global supply networks. The digitalisation of healthcare and the wider use of telemedicine are increasingly recognised as practical ways to contain emissions, largely by reducing the need for travel and some on-site activities. Several studies reviewed by Braithwaite et al. (56) and Pickard et al. (59) show that virtual consultations can meaningfully lower transport-related emissions while maintaining acceptable clinical outcomes and patient experience, and that reorganising follow-up pathways or relocating certain services closer to communities can further limit unnecessary mobility. When combined with improved scheduling, better logistics, and attention to equity in access to digital services, these measures offer a concrete and feasible contribution to the broader decarbonisation of health systems.
- Healthcare organisations should explore alternative transport options, such as electric or hybrid vehicles, and implement more efficient travel planning strategies
- Engage suppliers in emission reduction initiatives: healthcare institutions should collaborate with suppliers to implement joint programs aimed at reducing carbon footprints (e.g., Net Zero Supplier programs like those in the NHS) and those outlined in the WHO guidance on decarbonizing supply chains (15).
- Reduce food waste, promote sustainable nutrition, educate and raise awareness, increase plant-based options.

### 6.5 Climate adaptation and health system resilience

Adaptation actions must focus on strengthening the resilience of health systems to climate-related shocks while maintaining essential services under changing conditions.

- Conduct regular climate and health risk assessments to identify vulnerabilities in healthcare infrastructure, workforce capacity, and service delivery.
- Develop and implement comprehensive climate adaptation plans, including protocols for climate-induced health emergencies (e.g., heatwaves, floods) and updates to preparedness and emergency plans.
- Adopt proactive and reactive strategies at different levels (government, economic and finance, knowledge and behavioural change, physical and technological, nature-based solutions) to address climate shocks and stressors.
- Invest in climate-resilient infrastructure designed to withstand climate-related stresses, ensuring the continuity of essential healthcare services.
- Train staff on climate-related health risks and the response actions required during extreme weather events. Providing psychological support for staff after extreme events is also crucial for maintaining workforce resilience.

### 6.6 Training, communication, and public engagement

A knowledgeable and engaged workforce and community are essential to implementing climate action in healthcare systems.

- Develop training programs for healthcare staff to raise awareness of the health impacts of climate change and to equip them with the skills necessary to implement climate adaptation and mitigation strategies.
- Staff and community engagement in sustainability initiatives can drive significant change through daily practices and decision-making processes (60, 78). Engage communities in climate action by promoting health and sustainability education, and by involving patients and the public in initiatives to reduce healthcare sector emissions and improve health outcomes.

### 6.7 Health and equity co-benefits

Environmental sustainability in healthcare is not just about reducing emissions; it also creates significant co-benefits for health, including better air quality, healthier food systems, and more equitable health outcomes. Public health initiatives help constrain demand for health services, thereby minimising the environmental impact of healthcare facilities.

- Improve air quality: where healthcare decarbonisation reduces fossil-fuel combustion and associated air-pollutant emissions, it may contribute to lower population exposure and reduced cardiovascular and respiratory disease burden (61).
- Promote healthy, predominantly plant-rich dietary patterns with lower environmental impacts, which reduces the risk of diet-related chronic diseases such as obesity, diabetes, and heart disease (62).
- Encourage active mobility: supporting walking and cycling reduces emissions, increases physical activity and improves population health (63).
- Address health equity: a more sustainable and resilient healthcare system better protects vulnerable populations—such as the older adults, low-income groups, and migrants (64)—from climate impacts. By creating healthier environments and more equitable systems, these actions help reduce climate-related health disparities.
- Prevent unnecessary medical procedures: focusing on evidence-based treatments can prevent over-medicalisation, reducing healthcare emissions and improving care quality (65).
- Accessible, continuous and well-coordinated primary care is associated with lower rates of avoidable hospital admission for selected chronic conditions. OECD scenario analysis also suggests that shifting clinically appropriate care away from hospital settings could contribute to lower system emissions (66).

### 6.8 Indicators, monitoring and accountability

Lastly, to ensure that actions are effectively implemented and progress is made, robust monitoring and accountability mechanisms must be in place. In the context of healthcare decarbonisation, the development and implementation of monitoring systems with clearly defined, effective, reproducible, and scalable indicators at different levels of governance have become increasingly important. These systems not only allow emissions trends to be tracked over time and help identify the main sources of carbon emissions but also offer a means to assess whether the adopted measures are having the intended effects. A crucial aspect is that the indicator framework must be developed using a rigorous methodological approach, ensuring clarity of definitions, consistency of measurement, and reliability of data collection. At the same time, selecting indicators requires a careful balance between priority and feasibility: while it is essential to assess the areas of greatest impact, the monitoring and evaluation process should remain manageable for healthcare organisations, especially at the local level where operational capacities may vary. National and regional frameworks could provide a broader reference, but locally grounded monitoring tools enable the translation of strategic commitments into everyday practice and the timely identification of barriers. A coherent, methodologically sound monitoring architecture across all levels strengthens accountability and supports continuity and effectiveness in efforts to reduce emissions within the health sector.

- Develop and implement monitoring systems at national, regional, and local levels to track progress on climate action. This includes monitoring milestones, budgeting frameworks, and legally binding commitments, developing and tracking specific indicators to measure both emissions reductions and resilience improvements. The NHS in England has embedded mandatory “Green Plans” with transparent progress tracking.
- Establish regular evaluations of climate adaptation and mitigation plans to track impacts and identify areas for improvement, particularly in vulnerable subgroups.
- Italy currently lacks a centralised national framework to standardise measurement, compare regions, and incentivise improvement. Without shared metrics, regional fragmentation risks widening inequalities (67).

## 7 Best practices

Several international and national initiatives provide concrete examples of how health systems and institutions can operationalise climate mitigation, adaptation, and environmental sustainability. These best practices demonstrate the feasibility of translating strategic commitments into governance mechanisms, technical tools, and collaborative action.

### 7.1 System-wide leadership and accountability

The Greener NHS (23, 68) represents one of the most advanced examples of system-wide climate action in healthcare. The English National Health Service has established clear net zero targets by 2040 for emissions the NHS controls directly (Scope 1 and 2) and by 2045 for emissions the NHS can influence (Scope 3), supported by mandatory Green Plans for NHS organisations and a structured monitoring framework. This approach underscores the importance of clear targets, regulatory requirements, and accountability mechanisms in driving consistent action across a complex health system.

### 7.2 Energy transition and operational decarbonisation

At the subnational level, the Romagna Local Health Authority provides an example of effective implementation of energy efficiency and renewable energy measures within healthcare facilities. Its selection as a finalist for the 2024 EU Sustainable Energy Awards (40) highlights the potential for local health authorities to achieve measurable emissions reductions through integrated energy planning and infrastructure investment.

### 7.3 Practical tools and technical support

Several organisations support implementation by providing practical tools and technical guidance. HCWH Europe offers a range of resources to help health systems measure emissions, prioritise interventions, and implement low-carbon practices across clinical care, procurement, waste management, and food services (5). Similarly, the Canadian Coalition for Green Health Care provides tools, case studies, and guidance to support healthcare organisations reduce their environmental footprint and strengthen sustainability governance (26).

### 7.4 Cross-sectoral and interinstitutional collaboration

The Interinstitutional Group for Climate Neutrality in Bergamo illustrates the value of cross-sectoral collaboration at the local level. As part of Bergamo’s commitment to Net Zero Cities EU (69), the group brings together professional bodies, coordinated by the Local Medical Association, provincial health authorities, municipal government, academic institutions, and research organisations. This collaboration led to the development of the Handbook for Greener Healthcare Services, providing practical guidance tailored to healthcare settings (70). The group is also a finalist in the 2025 European Sustainable Healthcare Awards by HCWH, and it was presented at the 48th IHF World Hospital Congress in Geneva on November 11, 2025 (71).

Another example of local leadership is provided by the ATS di Brescia, which has launched a climate initiative to promote environmental responsibility within healthcare services. This initiative highlights the role of territorial health authorities in advancing sustainability through locally adapted strategies (72).

A third example of interinstitutional and intersectorial climate governance, in which public health authorities are playing a leading role, is represented by the nine Italian cities (Milano, Bergamo, Torino, Padova, Parma, Bologna, Prato, Firenze and Roma) that are committed to the European mission for climate neutrality by 2030, that is, 20 years ahead of the deadline set at COP 26 (73). These public healthcare providers, varying according to the areas of intervention defined by EuroCities (energy/buildings, transport/mobility, waste/wastewater, processes/products and governance), have designed and estimated the impact of a series of actions that will contribute to the goal of Net Zero Emissions by 2030. They have signed a Climate City Contract in 2023–24, along with other public and private partners in each area and will be subject to Local and European monitoring (74).

### 7.5 Capacity building and professional communities

Capacity building and knowledge exchange are essential enablers of sustained climate action. The European Climate Resilient Health Systems Course, hosted by Columbia University and ASPHER, offers a structured approach to training health professionals and policymakers on climate resilience and sustainability in health systems (75). In Italy, CERISMAS has established a community of practice focused on governance of socio-environmental sustainability in healthcare, facilitating peer learning and the dissemination of expertise across institutions (76).

## 8 Recommendations for Italy

To support the systematic transition towards environmentally sustainable and climate-resilient health systems in Italy, the following recommendations are proposed. Together, they aim to strengthen governance, accountability, knowledge exchange, and workforce capacity, while embedding sustainability into health system performance and service delivery. Given the decentralised and heterogeneous organisation of the Italian National Health Service, responsibilities cannot always be assigned exclusively to a single governance level. Most recommendations primarily require action at national and regional levels, while local health authorities and healthcare organisations play a central role in implementation, adaptation to local contexts and integration into organisational planning. The recommendations should therefore be interpreted as requiring coordinated and complementary action across different levels of the health system, rather than as applying uniformly to each level:

- Establish national coordination mechanisms, supported by regional mapping and formal membership structures, for organisations committed to environmentally sustainable healthcare. Specific actions, stakeholders, and roles should be formally included in Local Health Authority and healthcare organisation planning documents.
- Ensure access to adequate funding and the development of effective financing mechanisms to support the transition towards sustainability in hospitals.
- Integrate environmental sustainability indicators into the Essential Levels of Care (Livelli Essenziali di Assistenza, LEA) and healthcare performance evaluation systems, to ensure accountability and systematic monitoring.
- Promote the dissemination of good practices, including national and international experiences, technical guidance, training courses, and educational materials, and support the development of collaborative platforms to enable a self-sustaining network.
- Promote national and international professional exchanges across healthcare, public health, and technical profiles, to adapt existing tools and approaches to the Italian healthcare system.
- Strengthen research on healthcare quality and environmental sustainability, to support evidence-based policy development and implementation.
- Advocate for environmental sustainability and strengthen training for healthcare professionals, public health decision-makers, and citizens on climate change mitigation and adaptation.
- Support participation in international sustainability networks, including the Global Green and Healthy Hospitals initiative (77) and healthcare professional climate networks.
- Reduce avoidable hospitalisations by strengthening primary healthcare, health promotion, and prevention, generating co-benefits for population health, system efficiency, and environmental sustainability.

## 9 Conclusions: from roadmap to structural changes

The Italian roadmap is a welcome and forward-looking step. At present, however, it remains a high-level vision rather than a fully operational strategy. Compared with other countries, the Italian approach appears less binding, less systematically monitored, and less developed in areas such as primary care, research, and citizen engagement. To move from aspiration to transformation, Italy may benefit from institutionalising governance, financing, metrics, and participatory mechanisms so that sustainability becomes a structural and enduring dimension of the NHS rather than a temporary project. Italy could leverage European frameworks such as the EU Green Deal, the Recovery and Resilience Facility (RRF), or the National Recovery and Resilience Plan (PNRR) while they were active to support the ecological transition in healthcare. Aligning national health budgets with climate objectives would ensure long-term sustainability of investments. To make progress measurable, a robust monitoring and evaluation framework is needed. Defining a national set of sustainability indicators would enable systematic tracking over time. Research and innovation would also be key enablers of change. Universities, research institutes, and professional associations should be actively engaged in developing low-carbon technologies, evidence-based sustainability models, and tools for health impact assessment. Dedicated funding for research in sustainable healthcare would strengthen Italy’s capacity to generate and apply scientific evidence to policy. Furthermore, integrating sustainability and climate-health modules into education and training programs, both for current professionals and students, would foster a new generation of healthcare workers equipped to act as advocates for planetary health. Lastly, actions for sustainability in healthcare generate multiple co-benefits for both health and society. Measures such as promoting active mobility, improving air quality, sustainable nutrition, and reducing low-value care improve health outcomes while lowering emissions and costs. In conclusion, Italy’s roadmap lays a solid strategic foundation for aligning the National Health System with global sustainability and resilience goals.

A positive outcome of the Brazil’s COP30 was the Belém Health Action Plan, a framework structured around two cross-cutting principles and concepts: health equity and ‘climate justice’ and leadership and governance on climate and health with social participation. The Plan also outlines three lines of action for climate-resilient health systems:

- Surveillance and monitoring, focused on strengthening integrated and climate-informed health surveillance;
- Evidence-based policies, strategies, and capacity-building, aimed at enhancing the ability of national and local systems to implement effective, equity-driven solutions; and
- Innovation, production, and digital health, which promotes research, development, and access to technologies that meet the health needs of diverse populations.

To address the climate crisis, the Italian healthcare system should play an active role in the ecological transition while strengthening resilience and adaptation to extreme weather events. The actions outlined in this document represent a first step towards a more sustainable, resilient, and equitable National Health System, capable of protecting the health of present and future generations.

## Group member of SOSTERRETE Collaborative Group

Gabriella Abruzzo, Health Care Without Harm; Gloria Agazzi, Integrated University Hospital of Verona; Paola Angelini, Emilia-Romagna Regional Public Health Directorate; Stefania Barcella, Local Health Authority Papa Giovanni XXIII (Bergamo); Francesco Barone Adesi, University of Eastern Piedmont; Katsiaryna Bashlakova, Sapienza University of Rome; Sammy Bishop, NHS England; Antonio Bonaldi, Slow Medicine ETS; Giulia Bonanno, Sapienza University of Rome; Sofia Borghi, PoolMilano Engineering Society; Giuseppe Bortone, National Institute of Health (ISS), Italy; Roberta Bosco, University of Siena; Francesca Bravi, Local Health Authority Romagna; Serena Brunone, Sapienza University of Rome; Eva Cappelli, University of Verona; Maria Assunta Cappelli, University of Bolzano; Mario Carere, National Institute of Health (ISS), Italy; Luisa Carracciuolo, Local Health Authority Napoli 1 Centro; Angelo Cieri, Sapienza University of Rome; Walter Cristiano, National Institute of Health (ISS), Italy; Barbara Curcio Rubertini, University of Ferrara; Francesca De’ Donato, Department of Epidemiology, Lazio Regional Health Service; Manuela De Sario, Department of Epidemiology, Lazio Regional Health Service; Erica De Vita, University of Pisa; Benedetta Dellisanti, Sapienza University of Rome; Daniele D’Ettorre, Sapienza University of Rome; Chiara Di Blasi, National Institute of Health (ISS), Italy; Domenico Di Fazio, Sapienza University of Rome; Francesca Gorla, University Hospital of Bologna; Vittorio Grieco, National Institute of Health (ISS), Italy; Laura Grisolia, Sapienza University of Rome; Peppino Iannazzo, Sapienza University of Rome; Rachel Juel, National Institute of Health (ISS), Italy & Sapienza University of Rome; Ursula Kirchmayer, Department of Epidemiology, Lazio Regional Health Service; Paolo Lauriola, European Public Health Alliance; Jessica Lizzadro, Local Health Authority Ferrara; Camilla Lugli, National Institute of Health (ISS), Italy; Aurora Mancini, National Institute of Health (ISS), Italy; Laura Mancini, National Institute of Health (ISS), Italy; Maricia Mancino, University Hospital of Pisa; Marco Martuzzi, National Institute of Health (ISS), Italy; Roberta Monzani, Humanitas Research Hospital; Angela Nardin, National Institute of Health (ISS), Italy & University of Verona; Giovanni Nicolao, University of Campania; Lorenzo Nigi, Sapienza University of Rome; Lia Olivo, Catholic University of the Sacred Heart; Filippo Maria Panfili, Sapienza University of Rome; Romina Pausilli, Sapienza University of Rome; Alessia Pennimpede, Local Health Authority Torino 5; Simone Priolo, University Hospital of Verona; Ornella Punzo, National Institute of Health (ISS), Italy; Francesca Racioppi, World Health Organization; Laura Reali, ISDE Rome and Lazio; Chiara Reno, Local Health Authority Romagna; Francesco Romizi, International Society of Doctors for the Environment; Roberto Romizi, International Society of Doctors for the Environment; Gianluca Santini, Local Health Authority Papa Giovanni XXIII (Bergamo); Behjat Shokati, Sapienza University of Rome; Federica Tommasi, National Institute of Health (ISS), Italy; Chiara Tommasino, Sapienza University of Rome; Francesco Traglia, Migrant Health Unit, Local Health Authority Roma 1; Viola Turroni Casadei, Sapienza University of Rome; Sandra Vernero, Slow Medicine ETS; Giorgia Zanutto, Local Health Authority Bologna; Marianna Zarro, Sapienza University of Rome; Salvatore Zimmitti, University of Florence.
